# Distinct Microbiota Dysbiosis in Patients with Non-Erosive Reflux Disease and Esophageal Adenocarcinoma

**DOI:** 10.3390/jcm9072162

**Published:** 2020-07-08

**Authors:** Jerry Zhou, Prapti Shrestha, Zhiguang Qiu, David G. Harman, Wun-Chung Teoh, Sam Al-Sohaily, Han Liem, Ian Turner, Vincent Ho

**Affiliations:** 1 School of Medicine, Western Sydney University, Campbelltown, NSW 2560, Australia; 17046666@student.westernsydney.edu.au (P.S.); d.harman@westernsydney.edu.au (D.G.H.); qsohaily@hotmail.com (S.A.-S.); i.turner@westernsydney.edu.au (I.T.); v.ho@westernsydney.edu.au (V.H.); 2 Hawkesbury Institute for the Environment, Western Sydney University, Penrith, NSW 2750, Australia; g.qiu@westernsydney.edu.au; 3 Department of Gastroenterology, Campbelltown Hospital, Campbelltown, NSW 2560, Australia; wunchung.teoh@health.nsw.gov.au; 4 Nepean Hospital, Kingswood, NSW 2747, Australia; han.liem@health.nsw.gov.au

**Keywords:** microbiome, gastroesophageal reflux disease, proteome, non-erosive reflux disease, esophageal adenocarcinoma, Barrett’s esophagus, reflux esophagitis

## Abstract

Non-erosive reflux disease (NERD) and esophageal adenocarcinoma (EAC) are often regarded as bookends in the gastroesophageal reflux disease spectrum. However, there is limited clinical evidence to support this disease paradigm while the underlying mechanisms of disease progression remain unclear. In this study, we used 16S rRNA sequencing and mass-spectrometer-based proteomics to characterize the esophageal microbiota and host mucosa proteome, respectively. A total of 70 participants from four patient groups (NERD, reflux esophagitis, Barrett’s esophagus, and EAC) and a control group were analyzed. Our results showed a unique NERD microbiota composition, distinct to control and other groups. We speculate that an increase in sulfate-reducing Proteobacteria and Bacteroidetes along with hydrogen producer *Dorea* are associated with a mechanistic role in visceral hypersensitivity. We also observed a distinct EAC microbiota consisting of a high abundance of lactic acid-producing bacteria (*Staphylococcus*, *Lactobacillus*, *Bifidobacterium*, and *Streptococcus*), which may contribute towards carcinogenesis through dysregulated lactate metabolism. This study suggests the close relationship between esophageal mucosal microbiota and the appearance of pathologies of this organ.

## 1. Introduction

The prevalence of gastroesophageal reflux disease (GERD) has increased during the past two decades to affect 10–20% of the Western population and 5% in Asia [1]. GERD has traditionally been approached as a spectrum disease, where progressive exposure of the distal esophagus to gastric content leads to the development of more severe symptoms, mucosal damage, and complications [2]. Along the GERD spectrum, non-erosive reflux disease (NERD) is at the mild end, progressing towards erosive reflux esophagitis (RE), Barrett’s esophagus (BE), and ultimately esophageal adenocarcinoma (EAC). However, there is limited clinical information to support this paradigm. For example, current data indicate that only 10% of NERD progress to RE [3]. Similarly, incidences of EAC development in individuals with chronic GERD is relatively low (3.2/100,000) [4], while, to date, neither medical nor surgical treatments for GERD and BE have convincingly shown to prevent development of EAC [5]. As such, there has been considerable interest to determine the underlying mechanisms of these reflux diseases.

Under normal circumstances, reflux into the esophagus is prevented by the anti-reflux barrier compositing of the lower esophageal sphincter (LES), the extrinsic crural diaphragm, and the supporting structures of the gastroesophageal flap valve. When these protective components are compromised, the deleterious effects are additive, resulting in increasing reflux events. The duration of reflux exposure is determined by the effectiveness of esophageal reflux clearance, of which peristalsis, salivation, and presence of a hiatus hernia are key determinants. Sensory mechanisms in the distal esophagus determine the relationship between reflux exposure and symptom generation [2]. In addition, a number of hormones modulate gastric acid secretion and coordinate LES function. Gastrin inhibits gastric emptying and is shown to simultaneously induce gastric acid secretion and LES constriction. Conversely, gastric acid inhibitors, secretin and neurotensin, also reduce LES tone [6]. In recent years, the human esophageal microbiota has become associated with reflux disease development. Studies using 16S rRNA gene sequencing have identified specific microbiota profiles associated with GERD. Yang et al. [7], in 2009, identified a shift towards Gram-negative bacteria population in RE and BE microbiome, while Blackett et al. [8] combined cultivation- and sequencing-based evaluation and found a highly abundant Gram-positive bacterial population within EAC biopsies. Recently, a Japanese study performed a unique test to quantify total bacterial loads by quantitative 16S rRNA gene PCR [9]. The study reported that the relative abundance of taxa (Proteobacteria, Firmicutes, Bacteroidetes, Fusobacteria, and Actinobacteria), rather than absolute bacterial loads, are likely more relevant to esophageal diseases.

To investigate causality and microbial dysbiosis in reflux disease pathogenesis, we aim to characterize the esophageal microbiota and underlying host mucosa proteome. Previous studies have characterized microbiota composition in esophageal mucosa in specific conditions within the GERD spectrum (RE and BE [7], and EAC [8]), however the microbiota of across the sequelae of acid reflux disorders remains unexplored. Given the progressive nature of GERD, we hypothesized that studying the esophageal mucosal microbiota and underlying host proteome across the GERD spectrum would provide insights into disease progression and possible mechanisms of pathogenesis.

## 2. Material and Methods

### 2.1. Study Design and Sampling

The collection of samples was approved by the Research Ethics Committee of the South West Sydney Local Health District (HREC/16/LPOOL/143; 09 June 2016), Western Sydney University (RH11759; 07 July 2016), Nepean Blue Mountains Local Health District (SSA/18/NEPEAN/35 01 March 2017). Written consent was obtained from the participants.

Samples for analysis came from patients undergoing routine diagnostic upper gastrointestinal endoscopy for the investigation of symptoms at Campbelltown Hospital (Sydney, NSW, Australia) between 2017 and 2018. A total of 70 subjects were recruited and classified into 1 of 5 phenotypes based on symptomatic and histopathologic characteristics (Table 1): (1) controls (*n* = 16) were selected from individuals referred to endoscopy for investigation of iron deficiency anemia or lower abdominal pains who had no endoscopic evidence of esophageal, gastric, or duodenal diseases. A mucosal biopsy was retrieved and confirmed to be histologically normal in control samples. A simple GERD symptom questionnaire was used: patients were asked about the presence and frequency of six specific symptoms (heartburn, regurgitation, epigastric or chest pain, epigastric fullness, dysphagia, and cough) experienced over the last 3 months. Control patients did not experience any symptoms. (2) NERD participants (*n* = 11) had no endoscopic evidence of esophageal disease and normal histology but had experienced reflux symptoms within the last 3 months. (3) RE participants (*n* = 20) had endoscopic evidence of esophagitis based on the Los Angeles classification. (4) BE participants (*n* = 17) had at least 1 cm of columnar-lined esophagus and presence of intestinal metaplasia confirmed on histology. (5) EAC participants (*n* = 6) were recruited from Campbelltown Hospital and Nepean Hospital (Sydney, NSW, Australia) between 2017 and 2018. EAC diagnosis was proven on histology. Samples were taken at the time of investigation. Patients were aged > 18 years and the exclusion criterion was use of medications that could disrupt the microbiota, namely antibiotics or probiotics within 3 months of the study.

Cytology brush (Cook Medical, Bloomington, IN, USA) samples and endoscopic mucosal biopsies were taken at the time of insertion to minimize contamination. Cytology brushes were capped and endoscopically inserted to the target site, where 3 repeat brushings over the site were taken, before brushes was recapped and removed. Two biopsies were taken from each patient, 1 cm above the demarcation line (squamocolumnar junction), or at the site of pathology. One biopsy was used for histology, the other biopsy and brush sample were placed in separate sterile tubes, snap frozen in liquid nitrogen at −80 °C, and transported frozen for laboratory analysis.

### 2.2. Esophageal Microbiome Analysis

Bacterial DNA extraction was performed using a Purelink Microbiome DNA Purification kit (Invitrogen, Carlsbad, CA, USA) as per the manufacturer’s instructions. Bacterial dsDNA purity was determined by a NanoPhotometer and concentration by a Qubit dsDNA broad range assay kit and Qubit Fluorometer 2.0 (Invitrogen). Qualified DNA samples were sent to the Australian Genome Research Facility (AGRF, Sydney, NSW, Australia) for diversity profiling. Amplicon sequencing was performed targeting the hypervariable region (V1–V3, 27F/529R) of the 16S rRNA gene on the Illumina MiSeq platform (Illumina, San Diego, CA, USA), using the Illumina Nextera XT Index with paired-end sequencing. Raw paired-end Illumina reads were trimmed using Cutadapt [10]. Sequence analysis was performed using Quantitative Insights into Microbial Ecology 2 (QIIME Version 8.0.1623) [11]. A total of 8,457,356 raw sequence reads were obtained, and quality filtering resulted in 3,221,362 reads. Sequences were then clustered into operation taxonomic units (OTUs) following the default QIIME2 pipeline with referencing to 99% sequence similarity against the Greengenes database, version 13.8 [12]. Alpha diversity metrics included observed OTUs, Chao1, and Shannon index. Beta diversity was analyzed based on Bray–Curtis and Jaccard distances. Permutational multivariate analysis of variance (PERMANOVA [13]) and permutational multivariate dispersion (PERMDISP) analysis were performed in PRIMER version 6 (PRIMER-E, UK) [14]. Visualization was performed using non-metric multidimensional scaling (nMDS). Comparisons of relative abundance at the taxonomy levels across different groups were performed using multivariate generalized linear models (GLM) assuming a negative binomial distribution in R package “mvabund” [15], and significant pairs were identified using the pairwise Mann-Whitney test.

### 2.3. Esophageal Proteome Analysis

Biopsies were dissolved in buffer solution containing protease inhibitor cocktail 2% (*v*/*v*) (Roche Australia, Sydney, NSW, Australia), RapiGest SF (0.1% *w*/*v*) (Waters, MA, USA), and 75 mM aqueous ammonium bicarbonate. The cell suspension was disrupted through repeat freeze–thaw cycles in liquid nitrogen and a 50 °C water bath. The lysate mixture was heated at 95 °C for 1 h, followed by repeat freeze–thaw cycles. Protein concentration in lysate was quantified using a bicinchoninic acid assay kit (ThermoFisher Scientific, Sydney, NSW, Australia) as per the manufacturer’s instructions.

Reduction was accomplished with 5 mM dithiothreitol (Calbiochem, Kenilworth, NJ, USA) and alkylation with 15 mM iodoacetamide (Merck, Kenilworth, NJ, USA). Protein samples were digested at a protein:enzyme mass ratio of 100:1 (*w*/*w*) with mass spectrometer grade trypsin (Promega Gold, Madison, WI, USA) at 37 °C overnight. The reaction was stopped with 0.4% (*v*/*v*) aqueous trifluoroacetic acid (TFA) and lipids were removed from peptides through solid phase extraction on Oasis Hydrophilic-Lipophilic Balance (HLB) 30 mg, 1 mL cartridges (Waters Corp., Milford, MA, USA). Samples were washed with 0.1% (*v*/*v*) TFA and ultrapure water. Peptides were eluted with 70% (*v*/*v*) aqueous acetonitrile. Solvents were evaporated using a rotational vacuum concentrator before 0.1% aqueous formic acid was added. Following 10 min sonication and centrifugation, supernatants were transferred to Total Recovery (Waters) chromatographic vials for analysis. LC-MS/MS analysis was undertaken using a Waters nanoAcquity UPLC and Waters Xevo QToF mass spectrometer. LC-MS/MS analysis of tryptic digests was undertaken by injection of 3 μL of sample solution, loaded at 5 μL/min, onto the trapping column. The sample was desalted at the following solvent composition 1% acetonitrile + 0.1% formic acid (solvent B) in water + 0.1% formic acid (solvent A). The peptides were washed off the trap at 400 nL/min onto the analytical column using the following 60 min ramped method: after one minute, the initial solvent composition of 1% B was ramped linearly to 50% B by 31 min. A further linear ramp over two min to 85% B was immediately commenced. This composition was held from 33 to 36 min, at which time the solvent composition was returned to initial conditions. Following separation, the peptides were analyzed by tandem mass spectrometry in continuum mode, using the following instrument conditions: capillary voltage 2.3 kV, cone voltage 25 V, extraction cone 4 V, source temperature 80 °C, cone gas (N2) flow 20 L/h, nanoflow gas 0.50 L/h, purge gas 100 L/h, detector voltage 2350 V. Mass accuracy was maintained by lockmass correction, achieved by infusion at 0.5 µL/min of a solution of 200 ng/mL leucine encephalin in 50% aqueous acetonitrile plus 0.1% formic acid. A Data-Dependent Acquisition (DDA) experiment was performed which continuously scanned for peptides of charge state 2+ to 4+, with an intensity of more than 50 counts/s over the range *m*/*z* 350–1500. The three most abundant ions satisfying these conditions were fragmented for three seconds each, the resulting MS/MS spectra being collected over the range *m*/*z* 50–2000. The mass of each precursor peptide was then excluded for 30 s. Collision energies for peptide parent ions were ramped from 15 to 25 V at *m*/*z* 350 to 30–40 V at *m*/*z* 1500. The mass of the precursor peptide was then excluded for 30 s.

Data from three technical replicate experiments for each sample were analyzed qualitatively and quantitatively using the software Progenesis QI (Nonlinear Dynamics, Milford, MA, USA). Peak picking is performed where raw MS spectra data is reduced to a set of detected peptides and peptide ions. Protein identifications were obtained by exporting to MASCOT 2.6.00 (Matrix Science) with the embedded ion accounting algorithm of the software and searching a human database (UniProt KB/Swiss-Prot Protein Knowledgebase release 2018_10 of 20 October 2018). Variable modifications of carbamidomethyl (C), deamidated (NQ), oxidation (M), and proionamide (C) were used with peptide and MS/MS mass tolerances of 0.05 Da. For relative protein quantitation, the tandem mass tag (TMT) reporter ion intensities were extracted for each peptide. An isotopic purity correction was performed within Progenesis QI for each reporter based on the isotopic distribution of the sixplex-TMT reporters provided by the manufacturer. Statistical tests were performed using SPSS version 25 (IBM, Armonk, NY, USA), which included the Shapiro-Wilk test, Kruskal-Wallis H test, Mann-Whitney U test, and Levene’s test of homogeneity of variance. The STRING tool version 11.0 [16] (http://www.string-db.org) was used to construct the protein interaction networks and identify shared biological processes.

## 3. Results

### 3.1. Characteristics of the GERD and EAC Mucosal Microbiota

In total, 13 phyla, 87 genera, and 48 species-level taxonomic units were identified. Alpha diversity analysis showed that Chao1 richness estimator (Supplementary Figure S1) was significantly reduced in NERD compared to control (P_chao1_ = 0.041) and to RE (P_chao1_ = 0.022), while the Shannon diversity index showed no difference between groups. Beta diversity analysis was performed using Bray-Curtis and Jaccard distances (Supplementary Figure S2). The pairwise test (PERMANOVA) and test of dispersion (PERMDISP) identified significant differences between control and EAC diversity (P_ANOVA_ < 0.006, P_DISP_ = 0.030). Microbiota composition within each group is presented in Supplementary Figure S3.

### 3.2. Key OTUs Correlating with GERD Progression and EAC

**Figure 1:**
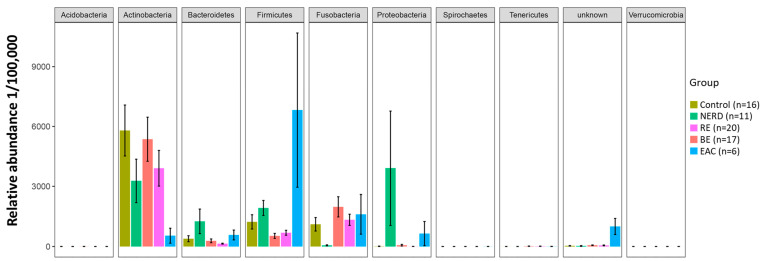
Differential operation taxonomic units (OTUs) at phylum level between non-erosive reflux disease (NERD), reflux esophagitis (RE), Barrett’s esophagus (BE), esophageal adenocarcinoma (EAC), and control groups.

**Figure 2:**
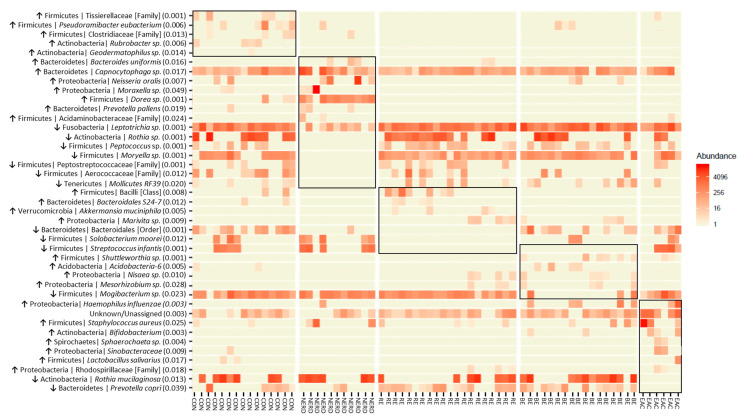
Heatmap representation of 41 differential operation taxonomic units (OTUs) between control (CON; *n* = 16) and disease phenotypes (non-erosive reflux disease (NERD; *n* = 11), reflux esophagitis (RE; *n* = 20), Barrett’s esophagus (BE; *n* = 17), and esophageal adenocarcinoma (EAC; *n* = 6)). OTUs from each disease phenotype were compared by multivariant analysis (anova.manyglm; *p*-value < 0.05). *p*-values are shown in brackets ( ), taxa classified above genus level are denoted in [ ]. Pairwise analysis identified differences in OTU associated with specific disease phenotype(s), these markers are grouped by black outlines. Trend-of-change for each marker within the phenotype is indicated with an arrow.

To identify candidate microbes of GERD and EAC pathogenesis, we examined differences in esophageal microbiota composition at different stages of GERD (from NERD to RE to BE) and in EAC. Multivariate analysis identified 41 differential OTUs within 9 phyla (Figure 1). Specific differential OTUs are presented in Figure 2 and significant sample pairs are summarized in Table 2.

The control microbiota had higher levels of Gram-positive Firmicutes and Actinobacteria compared to other groups. The NERD microbiota composition shifted towards Proteobacteria (*Neisseria oralis* and *Moraxella* sp.) and Bacteroidetes (*Bacteroides uniformis*, *Capnocytophaga* sp., and *Prevotella pallens*), and away from Fusobacteria (*Leptotrichia*) and Actinobacteria (*Rothia*). Several Firmicutes genus were reduced in NERD (*Peptococcus* and *Moryella*), while an increased abundance of *Dorea* resulted in an overall higher Firmicutes composition compared with control. The RE and BE microbiota were characterized by a shift away from Firmicutes (*Mogibacterium* sp., *Streptococcus infantis*, *Solobacterium moorei*) and towards Gram-negative Fusobacteria (*Leptotrichia* sp.) and Proteobacteria (Marivita, Nisaea, Mesorhizobium) relative to controls. The EAC microbiota was characterized by a shift towards Firmicutes, mainly *Staphylococcus aureus*, *Streptococcus infantis*, *Moryella* sp. and *Lactobacillus salivarius*, and Proteobacteria, while away from Actiobacteria (*Rothia mucilaginosa*) relative to controls.

### 3.3. Functional Alteration in GERD and EAC Host Mucosal Proteome

**Figure 3:**
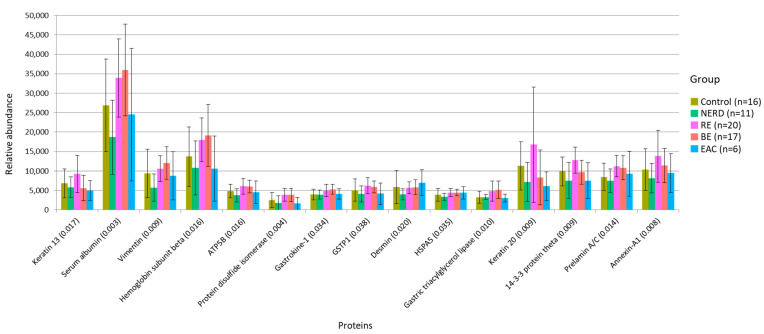
Protein abundance in 15 differential proteins between control and disease phenotypes are presented. *p*-values obtained by Kruskal-Wallis test are displayed in brackets ( ). Non-erosive reflux disease (NERD); reflux esophagitis (RE); Barrett’s esophagus (BE); esophageal adenocarcinoma (EAC); ATP synthase subunit beta (ATP5B); glutathione S-transferase *p* (GSTP1); Endoplasmic reticulum chaperone BiP (HSPA5).

**Figure 4:**
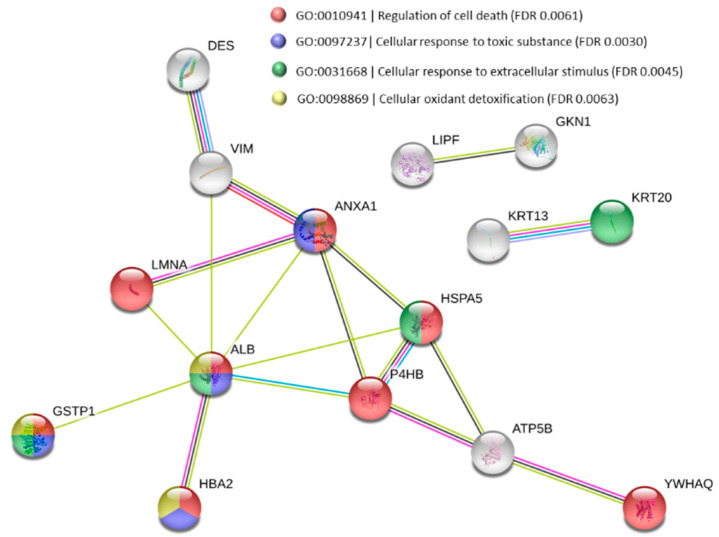
Protein–protein interaction map of 15 differential proteins between test groups. Three biological processes were characteristic of disease progression. Nodes are representative of protein species and lines represent protein–protein association. Nodes of the same color and/or joined by lines represents to a shared function. The STRING tool (http://www.string-db.org) was used to construct the interaction networks. Desmin (DES); vimentin (VIM); annexin A1 (ANXA1); prelamin-A/C (LMNA); serum albumin (ALB); glutathione S-transferase *p* (GSTP1); hemoglobin subunit beta (HBA2); Endoplasmic reticulum chaperone BiP (HSPA5); protein disulfide-isomerase (P4HB); ATP synthase subunit beta (ATP5B); 14-3-3 protein theta (YWHAQ); gastric triacylglycerol lipase (LIPF); gastrokine-1 (GKN1); keratin 13 (KRT13); keratin 20 (KRT20).

To gain mechanistic insights into the role of microbes and microenvironment in GERD and EAC, we conducted an unbiased, shotgun, quantitative proteomic analysis of endoscopic mucosal biopsies collected from the same region of the esophagus where microbiome brush samples were obtained. A total of 378 proteins were quantified; of these, only 26 proteins had a median ratio above 10-fold and >2 unique peptides. Fifteen (15) of the quantified proteins were identified as differentially expressed between test groups (Kruskal-Wallis test *p* < 0.05) (Figure 3). Significant group pairs were identified and are summarized in Table 3. Protein STRING analysis (Figure 4) identified a core group of proteins elevated in RE and BE samples associated with response to toxic substances: Annexin-A1, response to extracellular stimulus: Keratin 20, Endoplasmic reticulum chaperone BiP (HSPA5), and associated with oxidant detoxification: Serum albumin, Gastric triacylglycerol lipase (GSTP1), and Hemoglobin subunit beta. Several epithelial markers were elevated in RE, Keratin 13, Keratin 20, and Gastrokine-1, relative to other groups, while neoplasia markers desmin and vimentin were elevated in BE and EAC compared to control.

### 3.4. Effects of Proton Pump Inhibitor on Microbiota and Mucosal Proteome

Proton pump inhibitors (PPI) are a mainstay of reflux-disease treatment. A portion of NERD (36%), RE (50%), and BE (59%) subjects had undergone proton pump inhibitors (PPIs) treatment. Only 1 EAC subject was on PPI, hence there were insufficient numbers for comparative analysis. The effects of PPI treatment on microbiome composition and proteome abundance were determined by Wilcoxon test (*p* < 0.05) between PPI-treated and untreated subjects within each disease group. Alpha diversity analysis (Chao1 richness estimator and Shannon diversity index) did not show any difference between PPI-treated and non-treated groups. Beta diversity analysis was performed using Bray–Curtis and Jaccard distances. Pairwise test (PERMANOVA) and test of dispersion (PERMDISP) did not show significant difference between BE and PPI-treated BE diversity. Changes in specific bacteria taxa at the phylum level are presented in Supplementary Figure S4. PPI treatment is associated with an increase in Firmicutes and a decrease in Bacteroidetes and Proteobacteria composition.

The proteomic effects of PPI treatment showed reduced serum albumin and protein disulfide isomerase levels in NERD and RE treated with PPI compared with untreated, while these proteins were increased in BE treated with PPI compared to untreated (Supplementary Figure S5).

## 4. Discussion

In this study, we found distinct esophageal microbiota in patients with NERD, RE, BE, and EAC, providing insight into the molecular mechanisms of reflux-associated diseases and potentially new avenues of disease stratification. Our study found that the overall microbiota composition was altered in NERD and EAC compared with control, while unique OTUs present in NERD and EAC were not found in RE and BE patients. The reduction in proteins associated with external stimuli response in NERD and EAC further suggests that different molecular factors may be involved than those experienced by RE and BE.

Accumulating data support the characterization of NERD as a separate entity from other diseases in the GERD spectrum. Heterogeneity within NERD patients was speculated when this group responded less favorably to acid suppression therapy compared with RE patients [17]. This was later demonstrated through prolonged pH studies showing that 30–50% of NERD patients had normal acid exposure times but experienced reflux hypersensitivity [18,19]. A recent proteomic study proposed NERD and erosive-reflux disease to be distinct diseases, with NERD patients retaining capability to repair and regenerate esophageal mucosa following acid-pepsin insults, while erosive-reflux patient have reduced capability [20]. Here, we demonstrated the NERD microbiota to deviate from the GERD microbiota paradigm [7] of increased Gram-negative bacteria (Fusobacteria and Proteobacteria) and reduced Gram-positive Firmicutues (*Streptococcus*) observed in our RE and BE microbiota. Our proteome analysis also shows no significant alterations in NERD proteins compared with control, while in RE, elevated levels of stress response proteins were observed. The majority of our NERD patients (64%) were taken off proton-pump inhibitors due to a lack of response; therefore, we postulate that the unique NERD microbiota may be associated with reflux hypersensitivity. The role of microbiota in the bidirectional cross talk between the gut and brain is still far from being established. A recent study in irritable bowel syndrome (IBS) has shown that the transfer of fecal microbiota from hypersensitive IBS patients to germ-free rats is accompanied by the transfer of visceral hypersensitivity in the absence of any mucosal abnormality and change in gut permeability [21]. The authors reported a dysbiosis characterized by a significant increase in Proteobacteria (including sulfate-reducing *Enterobacteriacaea* and *Desulfohalobioaceae*) in IBS mice and speculate the pronociceptive role of luminal hydrogen sulfide (H_2_S) to induce excitation of sensor nerves through activation of T-type channels. Recent evidence suggests that H_2_S acts on transient receptor potential ankyrin 1 (TRPA1) directly in colonic afferent neurons to enhance the nociceptive function [22]. Our NERD data also show a significant increase in Proteobacteria and Bacteroidetes (including *Neisseria*, *Prevotella,* and *Bacteroides* with sulfate-reducing capabilities in some species), along with higher abundance of hydrogen gas producer *Dorea* sp. Although the specific sulfate-reducing bacteria identified in IBS patients were not identified in the distal esophagus of our NERD patients, its distinct microbiota warrants further research into the mechanistic role of microbiota–host interplay.

Sustained gastric reflux followed by infiltration of inflammatory cells into the esophageal mucosa are the predominant microenvironmental characteristics of RE and BE. Under these conditions, our RE and BE microbiota showed the characteristic shift away from Gram-positive Firmicute species and towards Gram-negative Fusobacteria and Proteobacteria relative to controls. This is in agreement with past studies in RE and BE patients [7,23,24]. The healthy distal esophagus harbors predominantly oral-derived Firmicutes (70–87%). This is altered in RE and BE to resemble gastric microbiota, which consists of fewer Firmicutes (22–30%) and more Gram-negative bacteria [25]. Although this study did not evaluate the oral or gastric microbiota, it is likely that the esophagus is continuously exposed to transient colonizers from oral saliva and gastric refluxate. The changes in esophageal microbiota are reflective of the source of transient bacteria and microenvironment. In the case of RE and BE, increased exposure to gastric refluxate combined with the acidic microenvironment led to a shift towards gastric bacteria composition. There has been growing interest in gastric bacteria *Helicobacter pylori* and its role in reflux diseases. Eradication of *H. pylori* have coincided with increased prevalence of GERD and increased risk of BE. Although *H. pylori* composition were not significantly different between our groups, these bacteria are known to modulate several hormones (gastrin, ghrelin, and leptin) and may have an indirect effect on GERD [26], as well as the host metabolism [27] and other organs, such as the pancreas [28].

The RE and BE proteome showed a concerted effort to ensure cell survival through the increased expression of proteins responsible for DNA and protein repair (prelamin-A/C, protein disulfide-isomerase, and 14-3-3 protein theta), which is in line with past studies [20,29,30]. Proteins associated with stress response annexin-A1 and GSTP1 were also elevated. Annexin-A1 is considered a putative mediator of glucocorticoid immunosuppressive activity [31] and is associated with chronic inflammation in the esophagus [32], while the GSTP1 enzyme belongs to a supergene family of enzymes involved in protection of cells from oxidative stress. The GSTP1 enzyme is the most important form in the esophagus, with increased expression during acid reflux [33]. Prolonged exposure to gastric enzymes and bile acids can exacerbate esophageal mucosal injury, leading to metaplasia and neoplasia [34]. The gastric-specific enzyme gastric triacylglycerol lipase was increased in RE and BE along with the gastric epithelium marker gastrokine-1. Evidence of neoplasia in BE and EAC was characterized by reduced gastrointestinal epithelium markers keratin 13 and keratin 20 [35], relative to RE, while epithelial-to-mesenchymal transition markers vimentin and desmin were increased in BE and EAC respectively, compared to control.

PPIs are a mainstay of reflux-disease treatment. Alterations of the esophageal microbiome as a result of PPI-reduced gastric acidity have been explored in several studies and shown to play a significant role in shaping microbial populations [36,37,38,39]. Our findings suggest an association between PPI treatment with small reductions in Bacteroidetes and Proteobacteria and increased Firmicutes (Supplementary Figure S4), previously also reported by Amir et al. [39]. The effects of PPI treatment on the NERD and RE proteome showed reduced levels of protein disulfide isomerase and serum albumin associated with stress response and detoxication, respectively. However, in BE, PPI treatment resulted in increased levels of these proteins (Supplementary Figure S5). The connection between PPI and neoplastic progression in BE is controversial. On the one hand, reduction of esophageal acid exposure by PPI decreases inflammation and proliferation [40]. On the other hand, PPI therapy interferes with esophageal exposure to secondary bile acids, increases circulating gastrin levels, and induces COX-2 upregulation [41,42]. Our results suggest that PPI treatment may have a positive effect in the NERD and RE but may negatively impact BE. However, given that the primary aim of our study was not to investigate PPI treatment, future studies involving larger sample sizes and better control of variables (e.g., PPI treatment time, dosage) are required to evaluate the impact of PPI treatment in BE.

The EAC microbiota is less well defined than that of RE and BE. Early culture-based studies of esophagectomy specimens isolated from EAC did not observe any differences in organisms isolated in benign versus malignant tissue [43,44]. Blackett et al. employed a modern sequencing technique to identify a “U-shaped” trend with comparable Gram-positive composition in healthy controls and EAC, distinct from GERD microbiota [8]. A study using Cytosponge sampling found Firmicutes genera *Lactobacillus* and *Streptococcus* dominant in EAC [45]. The authors propose that *Streptococcus* species are capable of surviving in a low nutrient microenvironment through competitor growth inhibition, a process which can induce host toll-like receptors and tissue damage by toxin release. Our data also showed similar EAC microbiota composition, consisting of *Staphylococcus aureus*, *Lactobacillus salivarius, Haemophilus influenzae, Sphaerochaeta* sp.*,* and *Bifidobacterium* sp., along with a rise in *Streptococcus infantis,* a species present in control and NERD but absent in RE and BE. The high abundance of lactic acid-producing bacteria (*Lactobacillus*, *Bifidobacterium*, *Staphylococcus*, *Streptococcus*) is traditionally associated with gastrointestinal health, given their beneficial roles in immune modulation [46], production of peroxides, acid, and bacteriocins, and also proteins that alter epithelial permeability and bind to intestinal receptors for pathogens [47]. However, in the context of cancer, elevated levels of lactic acid can be highly detrimental. Dysregulated lactate metabolism is one of the hallmarks of carcinogenesis [48]. Lactate can serve as an energy source in EAC and other cancers, inducing glycolytic enzymes, which leads to increase in ATP supply. This metabolite can also promote inflammation and stimulate tumor angiogenesis [49,50,51]. A consistent increase in abundance of lactic acid-producing bacteria (*Streptococcus*, *Lactobacillus*, *Bifidobacterium*) has been reported across different stages of gastric adenocarcinoma and is thought to directly promote carcinogenesis [52]. In vivo evidence for the role of lactic acid-producing bacteria in gastric carcinogenesis has been the colonization of an Insulin-Gastrin transgenic mouse model with lactic acid-producing microbiota, resulting in gastrointestinal intraepithelial neoplasia and upregulation of cancer-associated genes [53]. Other factors may also contribute towards the development of the EAC microbiota. Dysphagia, a common symptom of EAC, often reduces the patient’s dietary choices and relies more on liquid meals such as dairy-based nutrient formula. Dietary changes and subsequent availability of macronutrients can have a significant impact on gut microbiota composition [54], with a study showing increased *lactobacillus* composition in the distal esophagus of mice fed a high-fat diet [55]. Similarly, age-related changes in saliva production, given that the EAC cohort has the highest median age, can alter oral health and microbiota [56]. Reduced saliva production has been shown to effect gastrointestinal health, such as delayed healing of gastric and duodenal ulcers [57,58]. Reduced microbial diversity and relative abundance was also reported in the saliva of patients with EAC relative to controls [59]. A tentative connection between EAC and oral health is beginning to emerge in literature and may explain the origins of the distinct EAC microbiota.

A limitation of our study was that esophageal bacterial culture was not performed alongside 16S rRNA sequencing. Extracted DNA mainly comes from live bacteria, but this method also isolates DNA from dead bacteria, as well as bacterial residues. Hence, identification of DNA specific for any bacterial strains may not necessarily indicate the presence of live bacteria in the esophagus. Another limitation was the relatively few proteins identified from our biopsy tissue samples. Although our results were comparable with studies using similarly sized endoscopic biopsies [60], they were significantly fewer when compared with proteomic analysis using EAC surgical resections (>300 proteins) [30,61]. Small diagnostic mucosal biopsies at 3–5 mg [62] may not capture sufficient numbers of cancer cells for comprehensive proteomic analysis compared to much larger surgically resected tissue (30–60 mg) [61]. As a result, classical cancer markers such as epithelial cellular adhesion molecule (EpCAM) and inflammation markers were not identified in our EAC samples. Our results suggest that the complexity of esophagus proteome exceeds the analytical capacity of our approach. A more comprehensive proteomic approach may use deep tissue biopsies and enrichment of low abundance proteins by use of the solid phase hexapeptides ligand library.

Altogether, our results emphasize the importance of the microbial community and its interaction with the host in acid reflux-related diseases. We propose a central role for microbiota dysbiosis in NERD and EAC development. Although it is difficult to identify the mechanisms underpinning the role bacteria has in disease pathogenesis, the role of microbiota in promoting visceral hypersensitivity and development of adenocarcinoma has been demonstrated across other organs in the gastrointestinal tract. Therefore, our results further suggest the involvement of esophageal mucosal microbiota in NERD and EAC pathogenesis.

## Figures and Tables

**Table 1:** Subject characteristics.

Group	Control	NERD	RE	BE	EAC
Variables					
Subjects	16	11	20	17	6
Gender (male: female)	2:14	3:9	6:14	12:5	6:0
Median age (quartile 1–quartile 3)	52 (36–64)	63 (66–51)	55 (43–65)	58 (49–69)	67 (62–70)
Reflux symptoms	0	11 (100%)	18 (90%)	10 (59%)	3 (50%)
Reflux-medication					
Proton pump inhibitor	0	4 (36%)	10 (50%)	10 (59%)	1 (17%)
Inflammation/presence of lymphocytes	0	0	17 (85%)	4 (24%)	1 (17%)
Abnormal cell morphology					
Hyperplasia	0	0	7 (35%)	0	0
Metaplasia	0	0	0	16 (94%)	0
Dysplasia	0	0	0	1 (6%)	
Adenocarcinoma	0	0	0	0	6 (100%)

NERD: non-erosive reflux disease; RE: reflux esophagitis; BE: Barrett’s esophagus; EAC: esophageal adenocarcinoma.

**Table 2:** Significant pairs within the 41 differential operation taxonomic units (OTUs) between non-erosive reflux (NERD; *n* = 11), reflux esophagitis (RE; *n* = 20), Barrett’s esophagus (BE; *n* = 17), esophageal adenocarcinoma (EAC; *n* = 6), and control.

OTUs	*p*-Value	Distinct Group/s	Significant Pairs (*p*-Value < 0.05)
Firmicutes | Tissierellaceae [Family]	0.001	Highest in Control	NERD–Control (0.027); RE–Control (0.002); BE–Control (0.011)
Firmicutes | Pseudoramibacter eubacterium	0.006	Highest in Control	RE–Control (0.001); RE–BE (0.031)
Firmicutes | Clostridiaceae [Family]	0.013	Highest in Control	RE–Control (0.005); BE–Control (0.012)
Actinobacteria | Rubrobacter	0.006	Highest in Control	NERD–Control (0.037); RE–Control (0.009); BE–Control (0.047)
Actinobacteria | Geodermatophilus	0.014	Highest in Control	RE–Control (0.022); BE–Control (0.044)
Bacteroidetes | Bacteroides uniformis	0.016	Highest in NERD	Control–NERD (0.026); BE–NERD (0.016)
Bacteroidetes | Capnocytophaga	0.017	Highest in NERD	RE–NERD (0.009); BE–NERD (0.023); BE–EAC (0.046)
Proteobacteria | Neisseria oralis	0.007	Highest in NERD	RE–NERD (0.004); EAC–NERD (0.008)
Proteobacteria | Moraxella	0.049	Highest in NERD	BE–NERD (0.025)
Firmicutes | *Dorea*	0.001	Highest in NERD	Control–NERD (0.001); RE–NERD (0.001); BE–NERD (0.001); EAC–NERD (0.001); RE–Control (0.016)
Bacteroidetes | Prevotella pallens	0.019	Highest in NERD	RE–NERD (0.017); BE–NERD (0.014)
Firmicutes | Acidaminobacteraceae [Family]	0.024	Highest in NERD	BE–NERD (0.047); RE–NERD (0.047)
Fusobacteria | Leptotrichia	0.001	Lowest in NERD	NERD–Control (0.001); NERD–RE (0.001); NERD–BE (0.001); NERD–EAC (0.005)
Actinobacteria | Rothia	0.001	Lowest in NERD	NERD–Control (0.002); NERD–RE (0.001); NERD–BE (0.007); NERD–EAC (0.029)
Firmicutes | Peptococcus	0.001	Lowest in NERD	NERD–Control (0.012); NERD–RE (0.001); NERD–BE (0.002); NERD–EAC (0.027)
Firmicutes | Moryella	0.001	Lowest in NERD	NERD–Control (0.001); NERD–RE (0.001); NERD–BE (0.001); NERD–EAC (0.001)
Firmicutes | Peptostreptococcaceae [Family]	0.001	Lowest in NERD	NERD–Control (0.009); NERD–RE (0.004); NERD–EAC (0.036); BE–RE (0.002); EAC–RE (0.022); Control–BE (0.003)
Firmicutes | Aerococcaceae [Family]	0.012	Lowest in NERD	NERD–Control (0.002); NERD–RE (0.022); EAC–Control (0.011)
Tenericutes | Mollicutes RF39 [Order]	0.020	Lowest in NERD	NERD–Control (0.012); NERD–RE (0.028); NERD–BE (0.008)
Firmicutes | Bacilli [Class]	0.008	Highest in RE	NERD–RE (0.013); BE–RE (0.002)
Bacteroidetes | Bacteroidales S24-7	0.012	Highest in RE	NERD–RE (0.012); BE–RE (0.010); BE–Control (0.030)
Verrucomicrobia | Akkermansia muciniphila	0.005	Highest in RE	Control–RE (0.011); NERD–RE (0.022); BE–RE (0.011)
Proteobacteria | Marivita	0.009	Highest in RE	Control–RE (0.022); NERD–RE (0.026); Control–BE (0.028)
Bacteroidetes | Bacteroidales [Order]	0.001	Lowest in RE	NERD–Control (0.003); RE–Control (0.001); RE–BE (0.002); RE–EAC (0.001)
Firmicutes | Solobacterium moorei	0.012	Lowest in RE	RE–Control (0.010); RE–NERD (0.004); RE–EAC (0.013)
Firmicutes | *Streptococcus* infantis	0.001	Low in RE/BE	RE–Control (0.012); RE–NERD (0.004); RE–EAC (0.002); BE–Control (0.015); BE–NERD (0.001); BE–EAC (0.001)
Firmicutes | Shuttleworthia	0.001	Highest in BE	Control–BE (0.004); NERD–BE (0.017); RE–BE (0.002); EAC–BE (0.003); NERD–EAC (0.007); Control–EAC (0.004)
Acidobacteria | Acidobacteria-6	0.005	Highest in BE	RE–BE (0.006); NERD–BE (0.035); RE–Control (0.015)
Proteobacteria | Nisaea	0.010	Highest in BE	Control–BE (0.009); NERD–BE (0.024); Control–RE (0.043)
Proteobacteria | Mesorhizobium	0.028	Highest in BE	Control–BE (0.022); NERD–BE (0.041)
Firmicutes | Mogibacterium	0.023	Lowest in BE	BE–Control (0.030); BE–NERD (0.014); BE–RE (0.012)
Proteobacteria | Haemophilus influenzae	0.003	Highest in EAC	Control–BE (0.013); RE–BE (0.004); BE–EAC (0.013); Control–EAC (0.015)
Unknown/Unassigned	0.003	Highest in EAC	Control–EAC (0.003); NERD–EAC (0.015); BE–EAC (0.002); RE–EAC (0.002)
Firmicutes | *Staphylococcus* aureus	0.025	Highest in EAC	Control–EAC (0.014); BE–EAC (0.010)
Actinobacteria | *Bifidobacterium*	0.003	Highest in EAC	Control–EAC (0.002); NERD–EAC (0.004); BE–EAC (0.001)
Spirochaetes | Sphaerochaeta	0.004	Highest in EAC	Control–EAC (0.025); NERD–EAC (0.033); BE–EAC (0.015); RE–EAC (0.020)
Proteobacteria | Sinobacteraceae	0.009	Highest in EAC	NERD–EAC (0.028); BE–EAC (0.014); RE–EAC (0.018)
Firmicutes | *Lactobacillus* salivarius	0.017	Highest in EAC	RE–EAC (0.038); BE–EAC (0.041)
Proteobacteria | Rhodospirillaceae [Family]	0.018	Highest in EAC	Control–EAC (0.018); NERD–EAC (0.037); RE–EAC (0.021)
Actinobacteria | Rothia mucilaginosa	0.013	Lowest EAC	EAC–Control; EAC–NERD (0.023); EAC–BE (0.032); EAC–RE (0.001)
Bacteroidetes | Prevotella copri	0.039	Lowest EAC	EAC–RE (0.003); EAC–BE (0.001); NERD–RE (0.030)

**Table 3:** Significant pairs within the 15 differential proteins between non-erosive reflux (NERD), reflux esophagitis (RE), Barrett’s esophagus (BE), esophageal adenocarcinoma (EAC), and control.

Protein	*p*-Value	Distinct Group/s	Significant Pairs (*p*-Value < 0.05)
Keratin 13	0.017	Highest in RE	BE–RE (0.002); EAC–RE (0.021); NERD–RE (0.039)
Serum albumin	0.003	Low in Control/NERD	NERD–RE (0.001); NERD–BE (<0.001); Control–BE (0.030)
Vimentin	0.009	Lowest in NERD	NERD–BE (<0.001); NERD–RE (0.007); Control–BE (0.013)
Hemoglobin subunit alpha	0.016	Low in NERD/EAC	NERD–RE (0.010); NERD–BE (0.010); EAC–BE (0.042)
ATP synthase subunit beta	0.016	Lowest in NERD	NERD–RE (0.003); NERD–BE (0.004)
Protein disulphide-isomerase	0.004	High in RE/BE	NERD–RE (0.006); NERD–BE (0.008); EAC–RE (0.011); EAC–BE (0.013); Control–BE (0.033); Control–RE (0.039)
Gastrokine-1	0.034	Low in Control/NERD	Control–BE (0.007); NERD–RE (0.015)
Glutathione S-transferase *p* (GSTP1)	0.038	High in RE/BE	NERD–RE (0.014); CON–RE (0.031); NERD–BE (0.034)
Desmin	0.020	Lowest in NERD	NERD–BE (0.004); NERD–RE (0.005); NERD–EAC (0.007); Control–EAC (0.011)
Endoplasmic reticulum chaperone BiP (HSPA5)	0.035	Lowest in NERD	NERD–BE (0.007); NERD–RE (0.008)
Gastric triacylglycerol lipase	0.010	High in RE/BE	Control–BE (0.004); CON–RE (0.012); NERD–BE (0.026)
Keratin 20	0.009	Highest in RE	BE–RE (0.008); NERD–RE (0.010); EAC–RE (0.017); BE–Control (0.047)
14-3-3 protein theta	0.009	Highest in RE	NERD–RE (0.002); EAC–RE (0.009); BE–RE (0.014)
Prelamin-A/C	0.014	Low in Control/NERD	NERD–RE (0.004); NERD–BE (0.010); Control–RE (0.017)
Annexin A1	0.008	Highest in RE	NERD–RE (0.009); Control–RE (0.010); EAC–RE (0.012)

## Supplementary Materials

The following are available online at https://www.mdpi.com/2077-0383/9/7/2162/s1, Figure S1: Alpha diversity analysis of control, non-erosive reflux disease (NERD), reflux esophagitis (RE), Barrett’s esophagus (BE), and esophageal adenocarcinoma (EAC) using Chao1 richness estimator and Shannon diversity index, Figure S2: Non-metric multidimensional scaling (nMDS) plot for Bray–Curtis dissimilarity (abundance and composition) analysis and Jaccard (presence/absence) analysis of control, non-erosive reflux disease (NERD), reflux esophagitis (RE), Barrett’s esophagus (BE), and esophageal adenocarcinoma (EAC), Figure S3: Microbiota phylum composition within each group: control, non-erosive reflux disease (NERD), reflux esophagitis (RE), Barrett’s esophagus (BE), and esophageal adenocarcinoma (EAC), Figure S4: Differences in bacterial phylum composition between proton pump inhibitor treatment in non-erosive reflux disease (NERD), reflux esophagitis (RE), and Barrett’s esophagus (BE) disease phenotypes, Figure S5: (A) protein disulfide isomerase and (B) serum albumin were found differentially expressed between proton pump inhibitor (PPI)-treated and untreated mucosal proteomic samples in non-erosive reflux diseases (NERD), reflux esophagitis (RE), and Barrett’s esophagus (BE).
