# Supplementary material for: Distinct Microbiota Dysbiosis in Patients with Non-Erosive Reflux Disease and Esophageal Adenocarcinoma
Source: J Clin Med. 2020 Jul 8;9(7):2162. doi: 10.3390/jcm9072162 (PMC7408827; doi:10.3390/jcm9072162)
Supplement: Supplementary file 1 [file jcm-09-02162-s001.pdf]

Supplementary Materials:

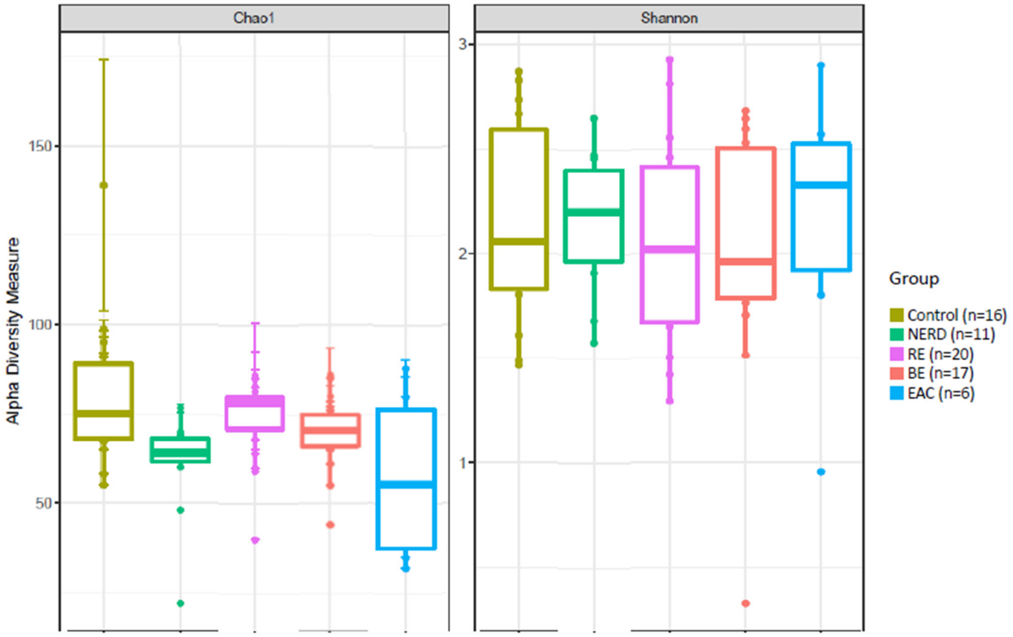

Figure S1. Alpha diversity analysis of control, non-erosive reflux disease (NERD), reflux esophagitis (RE), Barrett's esophagus (BE), and esophageal adenocarcinoma (EAC) using Chao1 richness estimator and Shannon diversity index.

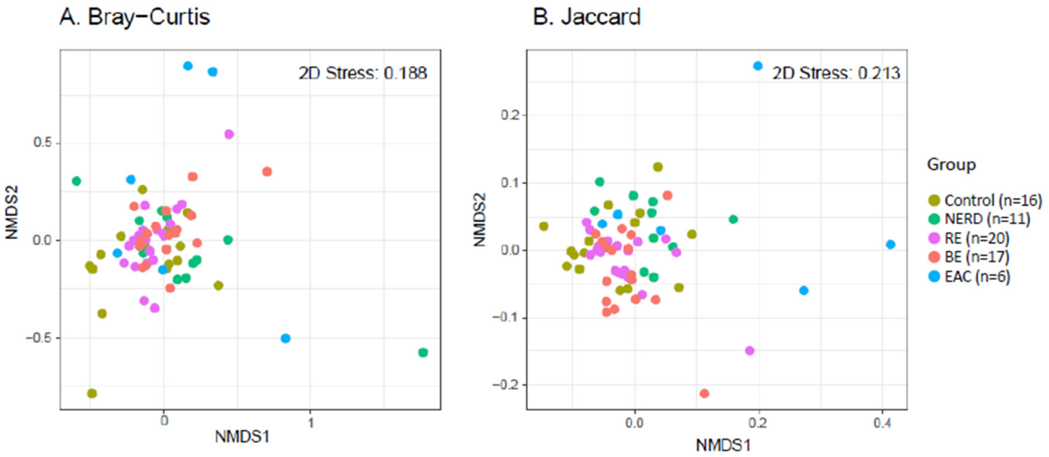

Figure S2. Non-metric multi-dimensional scaling (nMDS) plot for Bray-Curtis dissimilarity (abundance and composition) analysis and Jaccard (presence/absence) analysis of control, non-erosive reflux disease (NERD), reflux esophagitis (RE), Barrett's esophagus (BE), and esophageal adenocarcinoma (EAC).

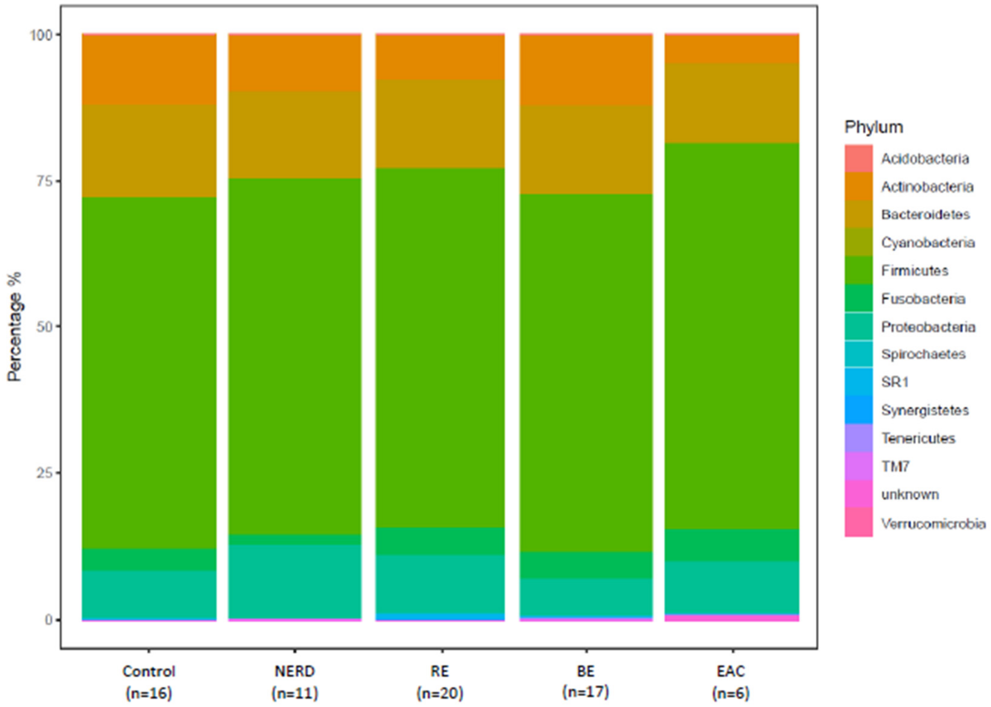

Figure S3. Microbiota phylum composition within each group: control, non-erosive reflux disease (NERD), reflux esophagitis (RE), Barrett's esophagus (BE), and esophageal adenocarcinoma (EAC).

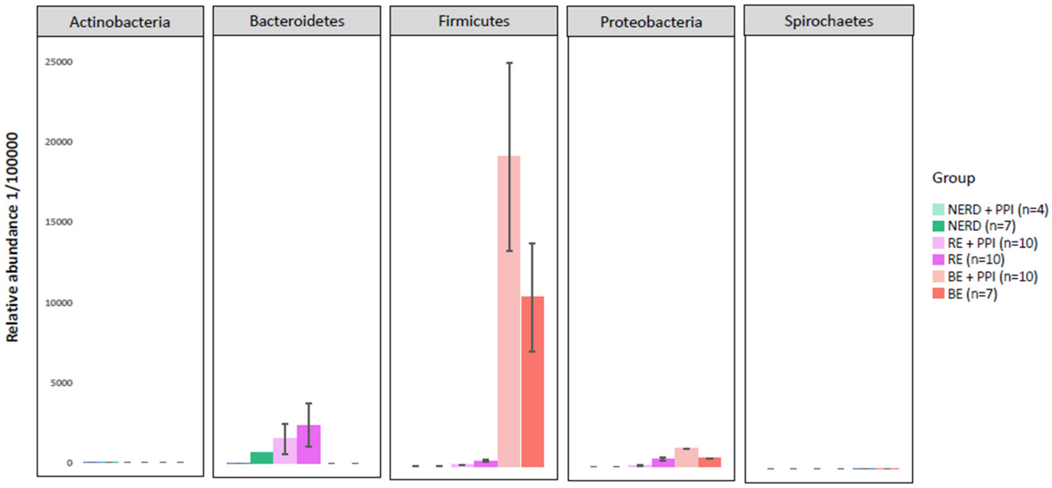

Figure S4. Differences in bacterial phylum composition between proton pump inhibitor treatment in non-erosive reflux disease (NERD), reflux esophagitis (RE), and Barrett's esophagus (BE) disease phenotypes.

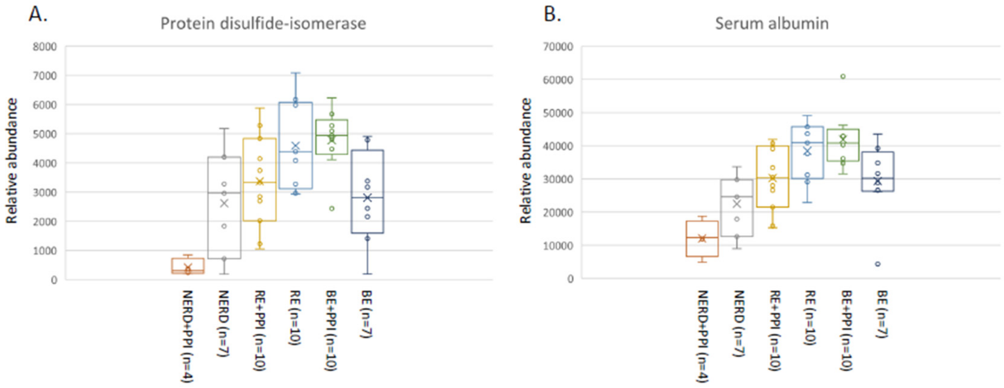

Figure S5. (**A**) protein disulfide isomerase and (**B**) serum albumin were found differentially expressed between proton pump inhibitor (PPI) treated and untreated mucosal proteomic samples in non-erosive reflux diseases (NERD), reflux esophagitis (RE) and Barrett's esophagus (BE).
